# Clinical evaluation of Emblica Officinalis Gatertn (Amla) in healthy human subjects: Health benefits and safety results from a randomized, double-blind, crossover placebo-controlled study

**DOI:** 10.1016/j.conctc.2019.100499

**Published:** 2019-11-27

**Authors:** Mahendra Parkash Kapoor, Koji Suzuki, Timm Derek, Makoto Ozeki, Tsutomu Okubo

**Affiliations:** a Taiyo Kagaku Co. Ltd., Nutrition Division, 1-3 Takaramachi, Yokkaichi, Mie, 510-0844, Japan; b Department of Molecular Pathobiology, Mie University, 2-174 Edobashi, Tsu City, Mie, 514 8507, Japan; c Suzuka University of Medical Sciences, Suzuka City, Mie, 510-0221, Japan; d Taiyo International Inc., Minneapolis, Minnesota, 55416, USA

**Keywords:** Amla (*Emblica Officinalis Gaertn*), Clinical trial, Vascular functions, Hematology, Lipid profile

## Abstract

The preventive efficacies and safety of *Emblica Officinalis Gatertn* (Amla), a most important and extensively studied plant in the traditional Indian Ayurvedic system of medicine, are presented. Eligible healthy adult subjects (n = 15) were randomized to receive either amla or placebo (500 mg per day) during an 18-week study. The efficacy parameters evaluated were the vascular function, blood hematology, oxidative and inflammatory biomarkers, glucose and lipid profiles, urinalysis, and liver hepatotoxicity. The amla intake showed significant improvements in the primary efficacy parameter of blood fluidity. There were also improvements in the secondary endpoints including lowering of von Willebrand factor (vWF), reduced 8-hydroxy-2′-deoxyguanosine (8-OHdG) as well as thrombin (TM) biomarkers of oxidative stress along with a significant improvement in HDL-cholesterol and lowering the LDL-cholesterol levels. No substantial changes were observed in liver hepatotoxicity, urinalysis, and hematology after consumption of amla compared to baseline or placebo. In addition, no adverse events, changes safety parameters or tolerance issues were observed after consumption of amla. In conclusion, amla supplementation showed acceptable palatability, improved endothelial functions and reduced oxidative stress.

## 1 Introduction

The metabolic syndrome is defined as having two of the following: hyperglycemia, hyperlipidemia, high blood pressure, low HDL-cholesterol, or abdominal obesity and has become an epidemic during the last few decades [1]. In recent years there has been a growing interest and awareness of the role of functional dietary supplements to help with the prevention of lifestyle-related diseases [2,3]. *Emblica Officinalis Gatertn*, commonly called amla, has traditionally been used for different medicinal purposes including: rheumatic pains, gonorrhea, asthma, hemorrhage, jaundice, dyspepsia, nausea, constipation, diarrhea, eye disease, brain health, intestinal ailments, diabetes mellitus, coronary heart diseases, and various cancers [4,5]. Modern science has shown amla to have hypoglycemic, anti-inflammatory, anti-hyperglycemic, *anti*-hyperlipidemic, and antioxidant properties in animal and human studies [[6], [7], [8]]. These properties may be due to the amla fruit containing high levels of vitamin C, tannins, polyphenols, fibers, minerals, proteins, and amino acids [4,9]. Standardized amla formulation usually contains the complex tannins and ellagitannins such as corilagin, geraniin, chebulagic acid, and elaeocarpusin, etc., which are the most active components and have high antioxidant activity [9]. Apart from the useful antioxidant activity, they have anti-thrombosis properties to promote vascular health by improve blood fluidity, anti-coagulant, and antiplatelet activity, which can cause a warming sensation. Amla also supports natural immunity and digestive functions. It is not completely understood, which amla components are responsible for each activity and they may be mediated through multiple different mechanisms [3,[10], [11], [12]]. The combined anti-inflammatory, anti-thrombosis, anti-coagulant, and anti-platelet activities of amla make it attractive target for the prevention of a variety of vascular disorders [13,14].

Further, there is limited evidence to support the longevity-promoting effects of *Emblica Officinalis*, but preliminary evidence suggests potent antioxidant activity that may explain these effects. In brain cells amla has high antioxidant activity [15]. Renal injury occurs when reactive oxygen species exceed the antioxidant reserve of renal tissue. Amla contains high concentrations of antioxidants ascorbic acid, gallic acid and phenolic compounds [16] suggesting that antioxidant activity of amla may be beneficial for the prevention of age-related renal disease and improvement of urinalysis parameters [7,17]. Also, amla extracts are reported to have the ability to modulate basal oxidative markers and enhance endogenous antioxidant defenses in a hepatocyte cell line (HepG2) [18]. Overall, amla has promise to help multiple systems due to its antioxidant activity.

The present randomized, placebo-controlled, double-blind crossover study sought to determine the preventive effect of amla on vascular functions, blood hematology, hemorheology, glucose and lipid profiles, oxidative stress and inflammatory biomarkers. Additionally, this study aimed to examine the safety profile of amla including urinalysis and hepatic risk parameters in healthy Japanese volunteers. An improvement in blood fluidity as the primary objective, and reduction of von Willebrand factor (vWF), 8-hydroxy-2′-deoxyguanosine (8-OHdG), and thrombin (TM) biomarkers of oxidative stress were secondary parameters along with a significant improvement in HDL-cholesterol and lowering the LDL-cholesterol levels were hypothesized.

## 2 Materials and method

### 2.1 Ethics statement

The study protocol was reviewed and approved by the Medical Ethical Committee of Mie University, Japan and registered (H-AO-00012006) at the hospital clinical trial registry network. Before participation, all participants gave written informed consent to the protocol approved by the Office of Research Promotion and Integrity of Mie University. Study-related procedures were carried out by the approved guidelines and ethical standards established in the Helsinki Declaration.

### 2.2 Subjects and criteria for their selection

Twenty four volunteers (n = 24) aged between 36 and 67 years were recruited to participate in the study and underwent screening measurements to determine eligibility for participation in the full protocol. Fifteen subjects (n = 15; M7, F8) were recruited after screening based on triglycerides levels, cholesterol levels, and the blood fluidity in whole human blood estimated using a microchannel array flow analyzer (MC-FAN; [19]. The following inclusion and exclusion criteria were used for the subjects to participate in the study. Healthy subjects with somewhat elevated blood triglycerides levels (>130 mg/mL to < 250 mg/mL), lower HDL cholesterol level (<54 mg/mL), and average values of blood fluidity (2.6–2.8 μL/s) were recruited for the study. Subject were excluded if (i) have an allergy and/or allergic disease including atopy, (ii) symptoms of cerebrovascular disorder, (iii) a history of myocardial infarction, (iv) atrial fibrillation and severe arrhythmia, (v) high renal dysfunction (serum creatinine >4.0 mg/dL), (vi) advanced liver function disorder, (vii) diabetes mellitus, (viii) anemia (hemoglobin <7 mg/dL), (ix) pregnant or lactating women, (x) consumption of medications or supplements affecting blood fluidity, (xi) other exclusions, judged by study medical practitioner during examination. Clinical characteristics of the subjects at baseline are presented in Table 1.

**Table 1:** Anthropometric physiological and vascular parameters of subjects of amla supplementation clinical trial.

Parameters	Placebo intake (mean ± sem)				Amla intake (mean ± sem)				ANCOVA (Among Groups)
	Before (W1, W2) or (W10, W11)	2 Weeks (W3, W4) or (W12, W13)	4 Weeks (W5, W6) or (W14, W15)	Post (3 Weeks) (W7,W9,W9) or (W16,W17,W18)	Before (W1, W2) or (W10, W11)	2 Weeks (W3, W4) or (W12, W13)	4 Weeks (W5, W6) or (W14, W15)	Post (3 Weeks) (W7,W9,W9) or (W16,W17,W18)	
Weight (kg)	70.6 ± 2.97	70.4 ± 3.03	70.4 ± 3.01	70.9 ± 2.87	70.6 ± 3.04	70.8 ± 2.98	70.4 ± 3.02	71.1 ± 3.00	2W: P = 0.42 4W: P = 0.89 Post: P = 0.51
		P = 0.31	P = 0.48	P = 0.44		P = 0.43	P = 0.70	P = 0.17	
BMI (kg/m^2^)	25.1 ± 0.63	25.1 ± 0.67	25.0 ± 0.63	25.2 ± 0.66	25.0 ± 0.64	25.3 ± 0.64	25.2 ± 0.62	25.3 ± 0.66	2W: P = 0.43 4W: P = 0.21 Post: P = 0.59
		P = 0.23	P = 0.47	P = 0.36		P = 0.47	P = 0.24	P = 0.18	
Body Fat (%)	30.5 ± 1.99	30.3 ± 1.93	30.2 ± 2.03	30.5 ± 1.97	30.2 ± 1.97	30.5 ± 2.04	30.9 ± 1.94	30.6 ± 1.97	2W: P = 0.29 4W: P = 0.038* Post: P = 0.39
		P = 0.52	P = 0.60	P = 1.0		P = 0.22	P = 0.007*	P = 0.18	
SBP (mmHg)	136.6 ± 8.02	148.4 ± 6.62	143.9 ± 5.82	148.4 ± 6.76	135.6 ± 8.53	134.11 ± 8.05	142.2 ± 5.23	147.9 ± 6.17	2W: P = 0.30 4W: P = 0.78 Post: P = 0.84
		P = 0.26	P = 0.50	P = 0.20		P = 0.42	P = 0.53	P = 0.29	
DBP (mmHg)	86.0 ± 5.03	93.0 ± 4.91	91.6 ± 4.35	91.9 ± 4.19	83.9 ± 4.85	84.7 ± 3.95	89.9 ± 4.26	94.4 ± 3.26	2W: P = 0.57 4W: P = 1.0 Post:P = 0.10
		P = 0.39	P = 0.50	P = 0.43		P = 0.43	P = 0.45	P = 0.15	
Pulse Rate (Beats/min)	68.9 ± 2.11	64.6 ± 2.10	65.7 ± 2.29	65.3 ± 2.29	72.7 ± 3.11	70.7 ± 3.94	65.0 ± 2.29	66.8 ± 2.86	2W: P = 0.64 4W: P = 1.0 Post: P = 0.69
		P = 0.27	P = 0.43	P = 0.39		P = 0.61	P = 0.18	P = 0.33	
Blood Fluidity (μL/sec)	2.77 ± 0.09	2.74 ± 0.07	2.43 ± 0.25	2.65 ± 0.10	2.63 ± 0.06	2.89 ± 0.09	2.89 ± 0.08	2.86 ± 0.19	2W: P = 0.012* 4W: P = 0.024* Post:P = 0.036*
		P = 0.81	P = 0.27	P = 0.41		P = 0.018*	P = 0.039*	P = 0.069	
Vascular Age (Waveform index)	−0.04 ± 0.15	−0.24 ± 0.09	−0.21 ± 0.16	−0.11 ± 0.13	−0.04 ± 0.11	0.15 ± 0.14	−0.12 ± 0.09	−0.24 ± 0.12	2W: P = 0.10 4W: P = 0.82 Post:P = 0.041*
		P = 0.33	P = 0.46	P = 0.70		P = 0.08	P = 0.56	P = 0.29	

### 2.3 Study design and sample collection protocol

**Fig. 1:**
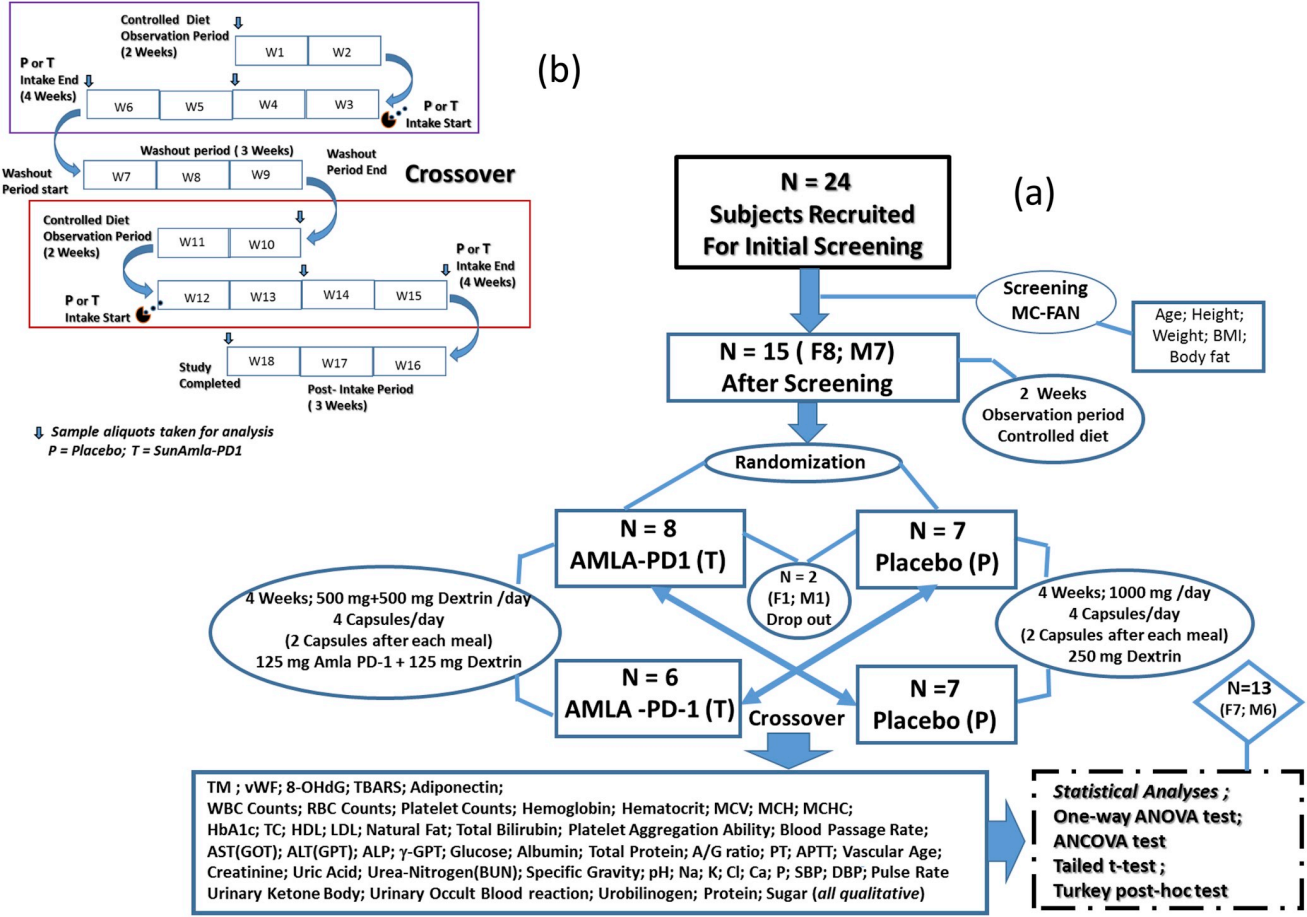
(a) Schematic presentation of the study protocol and procedures, and (b) Illustration of procedural details for evaluation of amla efficacies in randomized, double-blind, crossover placebo-controlled trial in healthy human subjects.

The study was a randomized, placebo-controlled, double-blind, crossover study (Fig. 1a), and a schematic illustration of the study profile is presented in Fig. 1b. Randomization was conducted using StatsDirect system by a researcher who was not involved in taking measurements and analysis (1:1; block randomization with random block size between 3 and 6). Subjects were randomly assigned into the amla or placebo group after two weeks (W1, W2) of controlled diet. No significant differences in the study parameters (age, height, body weight, body mass index (BMI), total fat mass, etc.) were observed between the treatment and placebo groups at baseline. The treatment group (T; n = 8) received amla capsules after each meal, while the placebo group (P; n = 7) received identical placebo capsules. After four weeks on the respective treatment (W3 to W6) a three-week washout period (W7 to W9) was implemented before crossover the other treatment. Two subjects (M1, F1) from decided to withdraw from the study due to personal reasons. After crossover, both groups consumed a controlled diet for two weeks (W10, W11) and then groups received the other treatment for four weeks (W12 to W15). At the end of the supplementation period, a post-intake period for three weeks (W16 to W18) was completed.

All subjects were asked to refrain from smoking, drinking alcohol or caffeine-containing beverages and excess consumption of coffee and tea (>2 cups/day) because caffeine may have a synergic effect with amla and the ability to modulate study parameters. All subjects were asked to abstain from any strenuous physical activity during the study. Subjects came to the laboratory in the morning after an overnight fast at the beginning of W1 where physical measurements (body weight, height, body fat, BMI, etc.), as well as fasting blood and urine samples, were conducted to determine baseline values. Before blood sample collection, a medical practitioner examined the subjects for the state of breathing as well as the presence or absence of epigastric/abdominal pains, diarrhea/constipation, vomiting, nausea, anorexia, and other subjective symptoms. Blood samples were drawn by a registered nurse in a seated position from an antecubital vein with anticoagulation by heparin solution after resting in a chair for at least 5 min.

Similarly, the fasted blood and urine samples were collected after at the end of W4, W6 and W9. Likewise blood and urine samples were collected at the beginning of W10 and then, at the end of W13, W15 and W18. Blood samples were snap-frozen in liquid nitrogen and stored at −80 °C until analysis.

### 2.4 Test supplementation and dosages

The commercial proprietary formulation of amla (SunAmla-PD1; Taiyo Kagaku Co. Ltd., Japan) as a dry powder was investigated in this study. It is prepared as a water extract of amla fruit pulp that has been hydrolyzed with pectinase followed by centrifugation with the supernatant spray dried. Study capsules were 250 mg hard gelatin capsules containing amla formulation (125 mg) and dextrin (125 mg), while placebo capsules were only dextrin (250 mg). Both supplements were identical in size and appearance. They are considered safe and well-tolerated at the reported dose. Subjects received (4 capsules/day) during both the treatment and placebo period. The total dose of amla was 500 mg per day (ellagitannins 1.2–1.5%; ellagic acid 0.1–0.2%; gallic acid, 1.5–2.0%; total polyphenols, 10–14%). The major active components of ellagitannins are corilagin, geraniin, elaeocarpusin, and chebulagic acid. Subjects were instructed to ingest two capsules every day immediately after breakfast and dinner during the four-week supplementation periods (W3 to W6) and (W12 to W15). Subjected were asked to comply with their supplementation and maintain a prescribed lifestyle during the study. A mandatory daily log of meal timing, including approximate calorie intake, supplementation intake, and total working, resting and sleeping hours were completed during the study.

### 2.5 Measured study parameters

The measured items were body temperature, pulse rate, blood pressure, vascular function, blood fluidity, hematological parameters, and biochemical parameters. Axillary body temperature was measured using a mercury thermometer of high precision. Automatic sphygmomanometer (Terumo; ES-P2000A) was used to measure blood pressure and pulse rate after resting at the sitting position at a stable position and height. Both systolic and diastolic blood pressures, body temperature, and pulse rate were measured three times consecutively, and the mean of the three measurements was recorded. Vascular age was measured using the acceleration pulse wave measurement system (Arnett PDU-M 100; Yumedica Co. Ltd. Osaka, Japan), and was conducted before the blood sample collection. The acceleration pulse waveform data as waveform index-I was measured twice, and the mean value of the waveform was used to estimate the blood vessel age. The measurement of blood fluidity as a primary efficacy parameter was done immediately after blood samples were drawn. Blood was collected in a separate tube for the measurement of blood passage time for the calculation of blood fluidity.

Hematological test parameters along with the specific tests of blood coagulation including Red blood cell count (RBC), White blood cell count (WBC), Hematocrit (Ht), Hemoglobin (Hb), Blood fluidity, Platelet counts (Plt), Platelet aggregation ability (PAA), Prothrombin time (PT), Activated partial thromboplastin time (APTT), Mean corpuscular hemoglobin (MCH), Mean corpuscular volume (MCV), Mean corpuscular hemoglobin concentration (MCHC) were measured by using an automatic cell counter (Beckman Coulter Co. Ltd.).

Other blood biochemical parameters including: GOT (AST), GPT (ALT), and γ–GTP (Randox kits), total protein (Biuret method), albumin (Bromocresyl green method), albumin/globulin (A/G ratio), blood glucose (Hexokinase glucose-6-pyruvate dehydrogenase method), triglycerides (Glycerin-1-phosphate-oxidase method), total cholesterol (Enzyme method), HDL-cholesterol (Homogeneous method), LDL-cholesterol (Friedewald equation), HbA1c (Chromatographic method), ALP (Spectroscopic method), uric acid (Enzymatic spectrophotometry method), total bilirubin (Microassay method), urea nitrogen (BUN) and creatinine (Enzyme method), Na, K, Ca, P (Flame photometry) and Cl (titration method) were measured. In addition, the secondary efficacy parameters thrombin receptor (TM), von Willebrand factor (vWF), 8-hydroxy-2′-deoxyguanosine (8-OHdG), thiobarbituric acid reactive substances (TBARS), adiponectin (ELISA kit) were also measured.

Urinalysis, a physical and chemical examination of the urine, was performed by collecting 5 mL of midstream urine by the subjects in a sample tube. A dipstick (test strips based on necessary wet microchemistry reactions) was used to detect the pH, specific gravity, urobilinogen, sugar, a ketone body, and occult blood. Qualitative and semi-quantitative results are expressed as either adverse or varying degrees of positive, indicating different amounts present.

### 2.6 Blood hemorheology assay using MC-FAN

Blood fluidity in whole blood collected from subjects was estimated using a microchannel array flow analyzer (MC-FAN; WBA-Neo, Tokyo, Japan). These results were employed for the initial screening and recruiting the subjects for this study. Briefly, the microchannel passage time for the 100 μL physiological saline as control was initially measured, followed by 100 μL heparinized blood obtained from the subjects. The revised values of the blood passage measure for the blood fluidity estimation was corrected and expressed as a function of the actual whole blood passage time over physiological saline solution passage time of 12 s at a pressure of 20 cm of water.

Further, platelet aggregation ability based on thrombus formation in blood treated with activating agent thrombin was determined by screen filtration pressure (SFC) detected using an aggregometer by measuring the decrease in flow rate as a function of agent concentration according to the manual of the manufacturer as described in Ref. [19]. Briefly, 200 μL of blood collected from subjects was mixed with 22 μL of thrombin at the concentration of 5U/mL of thrombin. The platelet aggregation ability was measured and expressed as % of inhibition against thrombin agonists.

### 2.7 Estimation of anticoagulant activity

Anticoagulant activities of the amla or placebo supplementation were determined by prothrombin time (PT) and activated partial thromboplastin time (APTT) assays. The assays were performed with 50 μL blood serum collected from the subjects added with 100 μL of PT or 50 μL of APTT and 50 μL of CaCl_2_ to measure coagulation time (sec) by a CA50 coagulometer (SYSMEX, Kobe, Japan) according to the manufacturer instructions.

### 2.8 Data processing and statistical analysis

All data values are expressed as means ± sem unless otherwise stated. Data analysis was performed by one-way analysis of variance (ANOVA) with study duration followed by Tukey's post hoc test, as well as using analysis of covariance (ANCOVA) to access the pre-post differences between both amla and placebo supplementations. All differences were considered significant at p ≤ 0.05. At the beginning of the study, a sample size of at least 12 subjects was estimated to be required to detect relevant physiological changes in study parameters between groups, at a 5% significance level with 90% power, assuming the drop out of 10%. Further, a post hoc power calculation was conducted to confirm the statistical power was adequate. An independent two-tailed Student's t-test was performed to verify the statistical significance.

## 3 Results

In accordance with the established criteria of subject selection based on blood rheology, we enrolled subjects with average values of blood fluidity (range 2.6–2.8 μL/s), because of the tendency for the blood fluidity to increase after test supplementations. The subjects who completed the study trial (n = 13; M6, F7) had a mean age of 51.9 ± 2.8 years.

### 3.1 Physiological and vascular parameters

Most of the physiological parameters changes with the time duration. Weight and BMI were in the normal range, and no significant differences were found. Also, no significant difference was observed for SBP, DBP, and pulse rate over the study. On the other hand, the blood fluidity was higher after the amla treatment compared to baseline. Significance differences by ANCOVA were observed in blood fluidity after two weeks (P = 0.012), four weeks (P = 0.024), and even after withdrawal (P = 0.036) of the amla treatment. Vascular age measured from the waveform index-I was only found to significant after the withdrawal of the amla intake (P = 0.041) compared to placebo (Table 1).

### 3.2 Hematological parameters and anticoagulant activity

The hematocrit, red blood cell, and white blood cell counts did not show significant changes during the placebo or amla period. The hemoglobin showed significant changes within both placebo and amla period compared to baseline, and also remained significant after the withdrawal of amla intake (P = 0.004). However, the hemoglobin levels were not significantly different between the amla and placebo periods. No significant difference was noticed in platelets counts for both placebo and amla intakes during the trial period, whereas compared to baseline the MCH and MCHC showed very significant changes within both placebo and amla intake duration (P < 0.001), and even after the withdrawal of the intake of both treatments (P < 0.05). Whereas, MCV showed the significance difference (P = 0.04) between both placebo and amla treatments at two weeks of supplementation (see Table 2).

**Table 2:** Changes in hematological and anticoagulant activity parameters in amla and placebo intake groups throughout the trial duration.

Parameters	Placebo intake (mean ± sem)				Amla intake (mean ± sem)				ANCOVA (Among Groups)
	Before (W1, W2) or (W10, W11)	2 Weeks (W3, W4) or (W12, W13)	4 Weeks (W5, W6) or (W14, W15)	Post (3 Weeks) (W7,W9,W9) or (W16,W17,W18)	Before (W1, W2) or (W10, W11)	2 Weeks (W3, W4) or (W12, W13)	4 Weeks (W5, W6) or (W14, W15)	Post (3 Weeks) (W7,W9,W9) or (W16,W17,W18)	
WBC (/μL)	5860 ± 419	5929 ± 480	5840 ± 383	5946 ± 419	5961 ± 383	5724 ± 452	6005 ± 471	6239 ± 530	2W: P = 0.52 4W: P = 0.92 Post: P = 0.67
		P = 0.86	P = 0.92	P = 0.81		P = 0.11	P = 0.87	P = 0.43	
RBC (x10^4^/μL)	484.7 ± 13.6	486.6 ± 12.8	480.5 ± 12.0	481.5 ± 13.8	481.3 ± 13.3	478.5 ± 12.5	475.5 ± 12.1	486.1 ± 12.8	2W: P = 0.38 4W: P = 0.78 Post: P = 0.24
		P = 0.59	P = 0.25	P = 0.35		P = 0.61	P = 0.31	P = 0.42	
Hemoglobin (g/dL)	14.6 ± 0.42	14.2 ± 0.39	14.1 ± 0.37	14.5 ± 0.42	14.7 ± 0.41	14.5 ± 0.38	14.3 ± 0.38	14.4 ± 0.41	2W: P = 0.56 4W: P = 0.79 Post: P = 0.23
		P = 0.05*	P = 0.015*	P = 0.80		P = 0.04*	P = 0.002*	P = 0.004*	
Hematocrit (%)	44.5 ± 1.21	44.5 ± 1.51	44.1 ± 1.09	44.1 ± 1.29	44.1 ± 1.21	44.0 ± 1.17	43.7 ± 1.12	44.6 ± 1.20	2W: P = 0.92 4W: P = 1.0 Post: P = 0.16
		P = 0.86	P = 0.20	P = 0.18		P = 0.84	P = 0.42	P = 0.39	
Platelets Count (x10^4^/μL)	28.4 ± 2.46	28.5 ± 2.41	28.1 ± 2.17	29.2 ± 2.55	28.9 ± 2.64	28.1 ± 2.45	28.3 ± 2.23	28.7 ± 2.53	2W: P = 0.48 4W: P = 0.82 Post: P = 0.46
		P = 0.92	P = 0.57	P = 0.37		P = 0.18	P = 0.47	P = 0.82	
PT (Sec)	11.6 ± 0.47	10.9 ± 0.17	11.9 ± 1.30	10.47 ± 0.12	11.6 ± 0.33	10.9 ± 0.23	10.3 ± 0.22	10.38 ± 0.29	2W: P = 0.11 4W: P = 0.01* Post: P = 0.013*
		P = 0.13	P = 0.87	P = 0.07		P = 0.025*	P = 0.007*	P = 0.008*	
APTT (Sec)	31.7 ± 1.13	32.6 ± 1.44	35.8 ± 2.28	32.3 ± 1.71	32.4 ± 1.58	32.3 ± 1.56	33.5 ± 1.75	31.9 ± 1.56	2W: P = 0.73 4W: P = 0.60 Post: P = 0.81
		P = 0.67	P = 0.15	P = 0.81		P = 0.59	P = 0.64	P = 0.83	
Platelet Aggregation (%)	96.7 ± 2.50	100 ± 0	100 ± 0	93.7 ± 5.04	86.5 ± 4.81	99.7 ± 0.28	94.0 ± 4.49	95.1 ± 2.51	2W: P = 0.30 4W: P = 0.20 Post: P = 0.28
		P = 0.095	P = 0.23	P = 0.33		P = 0.027*	P = 0.20	P = 0.16	
MCV (fl)	91.7 ± 0.98	92.0 ± 0.99	91.9 ± 0.97	91.7 ± 0.90	91.9 ± 1.07	91.5 ± 0.92	91.8 ± 0.90	91.7 ± 0.98	2W: P = 0.04* 4W: P = 0.15 Post: P = 0.52
		P = 0.16	P = 0.38	P = 1.0		P = 0.05*	P = 0.52	P = 0.37	
MCH (pg/cell)	30.4 ± 0.39	29.7 ± 0.32	29.7 ± 0.35	29.9 ± 0.27	30.3 ± 0.27	29.8 ± 0.31	29.7 ± 0.32	29.9 ± 0.36	2W: P = 0.57 4W: P = 0.48 Post: P = 0.89
		P ≤ 0.001*	P ≤ 0.001*	P = 0.003*		P ≤ 0.001*	P ≤ 0.001*	P = 0.017*	
MCHC (g/dL)	33.1 ± 0.21	32.5 ± 0.12	32.4 ± 0.16	32.6 ± 0.18	33.1 ± 0.15	32.3 ± 0.20	32.3 ± 0.15	32.6 ± 0.20	2W: P = 0.08 4W: P = 0.03* Post: P = 0.28
		P ≤ 0.001*	P ≤ 0.001*	P = 0.005*		P ≤ 0.001*	P ≤ 0.001*	P = 0.03*	

The platelet aggregation showed significant stepwise improvement within the amla intake (P = 0.027) after two weeks of supplementation, but no difference were observed between placebo and amla intake for platelet aggregation. Anti-coagulant activity parameter PT showed a statistically significant reduction (P < 0.01) for the amla intake throughout the trial and also after the post-treatment period. Further, a significant difference between the placebo and amla treatment groups after four weeks of supplementation (P = 0.01) and also after the post treatment period (P = 0.013) was observed. The anticoagulant activity parameter APTT showed no significant changes within and between the placebo and amla treatment groups.

### 3.3 Blood biochemical parameters and biomarkers

Fasting blood glucose levels significantly changed after four weeks (P = 0.03) and tended to after the withdrawal (P = 0.06) of amla intake. Both the fasting glucose and triglyceride levels remained in the normal range during the amla and placebo intake periods. No significant difference in HbA1c, triglycerides, and total cholesterol levels were observed during both the placebo and amla periods. HDL cholesterol levels were significantly higher after two weeks (P = 0.03) and remained higher than baseline values when comparing to the placebo or amla intakes. The LDL cholesterol levels decreased but did not reach statistical significance (P = 0.09) after two weeks of amla supplementation compared to placebo. The Ca, Na, K, P levels in the blood significantly changed with amla, while chloride levels significantly changed in both placebo and amla intake periods (see Table ST1; supplementary information).

**Fig. 2:**
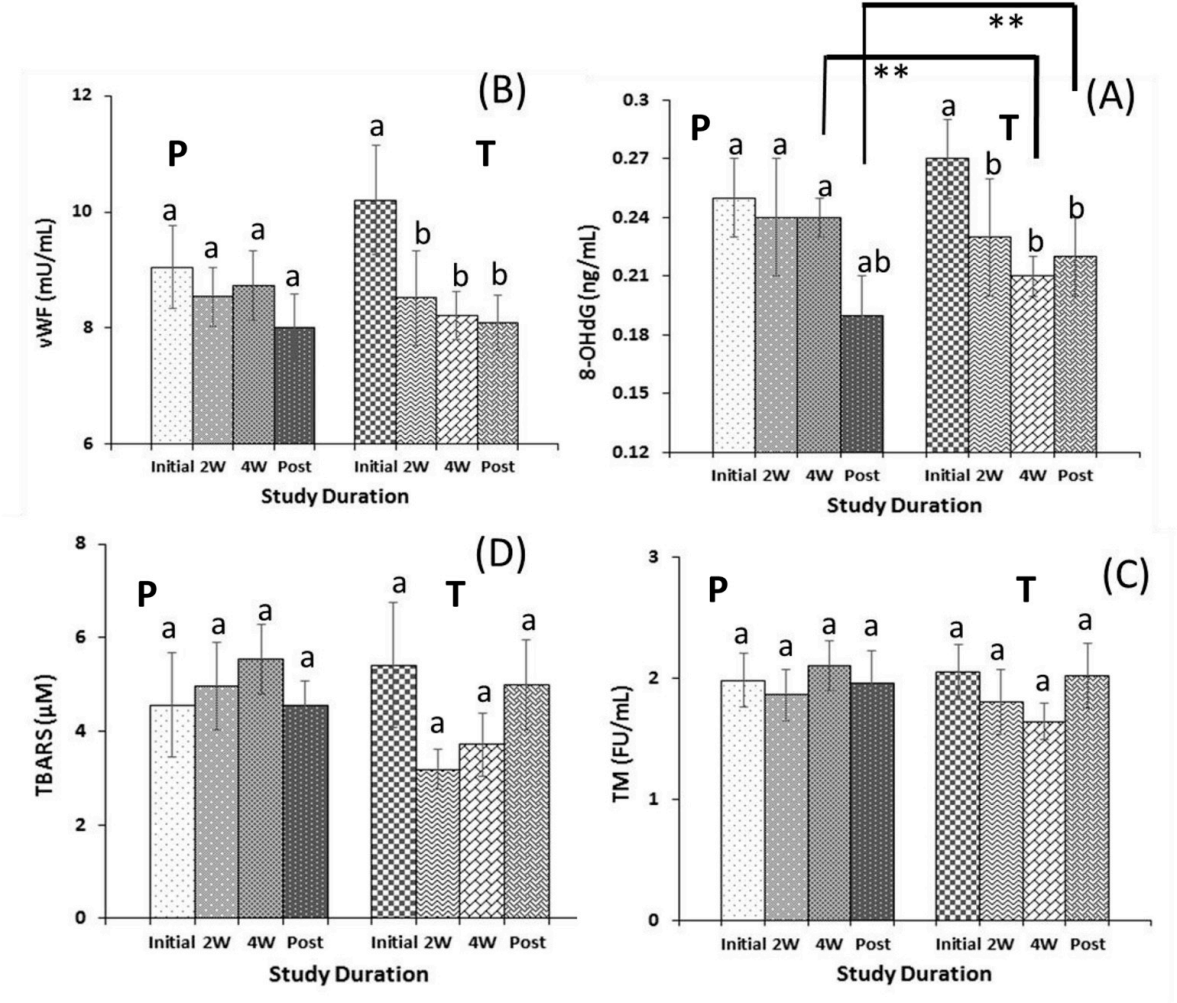
Illustration of oxidative and inflammatory biomarkers assay (A) 8-hydroxy-2′-deoxyguanosine; 8-OHdG, (B) von Willebrand factor; vWF, (C) Thrombin; TM and (D) Thiobarbituric acid reactive substances; TBARS during the amla supplementation duration. The result depicts the comparison between the groups (**a** to **b** denote significance of p-value p < 0.05; **a** to **ab** denote nearly significance of p-value p < 0.07; measured by one-way ANOVA), and among the groups (* denote significance of p-values p < 0.05; **denote significance of p-values p < 0.01; measured by ANCOVA).

Of secondary efficacy parameters, the concentration of 8-hydroxy-2′-deoxyguanosine (8-OHdG) was significantly decreased compared to baseline during the amla intake period and even after the withdrawal (P < 0.05) of amla supplementation. Further, compared to placebo, a significant difference (P < 0.01) was also observed after four weeks and even after post-intake of amla (Fig. 2a). Also, the von Willebrand factor (vWF) was significantly lowered (P < 0.05) during the amla intake period (Fig. 2b). While a non-significant decrease in the thrombin (TM) receptor concentration and thiobarbituric acid reactive substances (TBARS) was observed (Fig. 2c and d) and remained in the normal range during the amla intake period. No significant changes were observed in adiponectin (see Table 3).

**Table 3:** Assessment of characteristic oxidative and inflammatory biomarkers and their statistical comparison to review the potential of amla preventive effects among healthy human subjects.

Parameters	Placebo intake (mean ± sem)				Amla intake (mean ± sem)				ANCOVA (Among Groups)
	Before (W1, W2) or (W10, W11)	2 Weeks (W3, W4) or (W12, W13)	4 Weeks (W5, W6) or (W14, W15)	Post (3 Weeks) (W7,W9,W9) or (W16,W17,W18)	Before (W1, W2) or (W10, W11)	2 Weeks (W3, W4) or (W12, W13)	4 Weeks (W5, W6) or (W14, W15)	Post (3 Weeks) (W7,W9,W9) or (W16,W17,W18)	
TM (FU/mL)	1.98 ± 0.22	1.86 ± 0.21	2.10 ± 0.21	1.96 ± 0.26	2.05 ± 0.23	1.80 ± 0.27	1.64 ± 0.15	2.02 ± 0.27	2W: P = 0.67 4W: P = 0.16 Post: P = 0.86
		P = 0.68	P = 0.76	P = 1.0		P = 0.11	P = 0.12	P = 0.92	
vWF (mU/mL)	9.05 ± 0.71	8.54 ± 0.51	8.73 ± 0.60	8.02 ± 0.56	10.2 ± 0.94	8.52 ± 082	8.22 ± 0.41	8.09 ± 0.48	2W: P = 0.27 4W: P = 0.58 Post: P = 0.18
		P = 0.56	P = 0.75	P = 0.28		P = 0.011*	P = 0.045*	P = 0.056*	
TBARS (μM)	4.56 ± 1.11	4.96 ± 0.93	5.54 ± 0.73	4.55 ± 0.51	5.41 ± 1.33	3.18 ± 0.43	3.72 ± 0.67	4.99 ± 0.95	2W: P = 0.19 4W: P = 0.46 Post: P = 1.0
		P = 0.80	P = 0.54	P = 1.0		P = 0.14	P = 0.34	P = 0.80	
*8-OHdG (ng/mL)*	0.25 ± 0.02	0.24 ± 0.03	0.24 ± 0.01	0.19 ± 0.02	0.27 ± 0.02	0.23 ± 0.03	0.21 ± 0.01	0.22 ± 0.02	2W: P = 0.22 4W: P = 0.002* Post: P = 0.003*
		P = 0.77	P = 0.60	P = 0.06		P = 0.033*	P = 0.036*	P = 0.031*	
Adiponectin (ng/mL)	1.33 ± 0.19	1.47 ± 0.21	1.50 ± 0.21	1.51 ± 0.23	1.41 ± 0.23	1.32 ± 0.17	1.47 ± 0.19	1.40 ± 0.17	2W: P = 0.47 4W: P = 0.24 Post: P = 0.11
		P = 0.69	P = 0.63	P = 0.61		P = 0.86	P = 0.83	P = 1.0	

### 3.4 Evaluation of hepatic risk parameters

The test results of the hepatic factors, which are helpful to determine the liver functions are illustrated in Table ST2 (see supplementary information). During the amla and placebo period no difference in the levels of AST (GOT). The ALT (GPT) level was lower after two weeks (P = 0.02) of amla intake and remained at lower than baseline levels. Also, a lower ALP and γ-GPT levels were noticed during the trial periods, but were not statistically decreased. All critical hepatic risk parameters remained in the normal range throughout for both amla and placebo periods. Total bilirubin significantly decreased after two weeks of amla intake (P = 0.04) compared to baseline, while no significant differences were found in total protein, albumin, albumin/globulin ratio, creatinine, uric acid, and urea-nitrogen levels assays.

### 3.5 Urinalysis parameters

Urinalysis of the most common chemicals before and after the placebo and amla intake periods were collected. The pH range was slightly acidic (5.5–6.1) and the specific gravity range of urine was always less than 1.02 during the trial periods, but no significant changes were noted. Quantitative assay of urobilinogen showed normal levels for all subjects (n = 13) throughout the study. Quantitative protein and sugar levels were also normal except somewhat doubt (no immediate concern) was registered for one subject during the post-supplementation periods of placebo (protein, n = 1) and amla (sugar, n = 1), respectively. The placebo group had nine subjects with normal occult blood in urine and four subjects with some borderline doubt but in healthy condition (n = 9, A1-judgment; n = 4, A2-judgment) at baseline and remained unchanged during the placebo period. The amla intake group registered twelve subjects without abnormalities and one with no immediate concerns at baseline, however, eight subjects without abnormalities and five subjects with no immediate concerns were reported at the end of amla treatment possibly due to crossover nature of the study trial (see Table ST3; supplementary information).

### 3.6 Adverse events and examination opinions

No significant conditions or adverse events were found in the physician examination implemented on the first day of both study periods through the completion of the study. Some minor conditions were reported mainly during the placebo intake. One subject reported diarrhea feelings during the placebo intake of the trial. While, one subject reported for the loose bowel movements after two weeks, and other reported constipation after four weeks of placebo supplementation. Also, one subject reported cold after 2–4 weeks of placebo intake duration of the trial. One subject reported edema before the start of amla intake; however, no other conditions were reported during the amla intake period (see Table ST4; supplementary information).

Furthermore, no anomalies were observed in the subjective symptom surveys or daily activity diaries wherein the subjects recorded any concerning information during the study, such as unusual circumstances or the side effects. There was 100% compliance with the placebo and amla intakes during the trial for all subjects who completed this study. Also, the diet and meal intake were not noteworthy, and there were no subjects with eating and lifestyle habits that deserve special mention.

## 4 Discussion

**Fig. 3:**
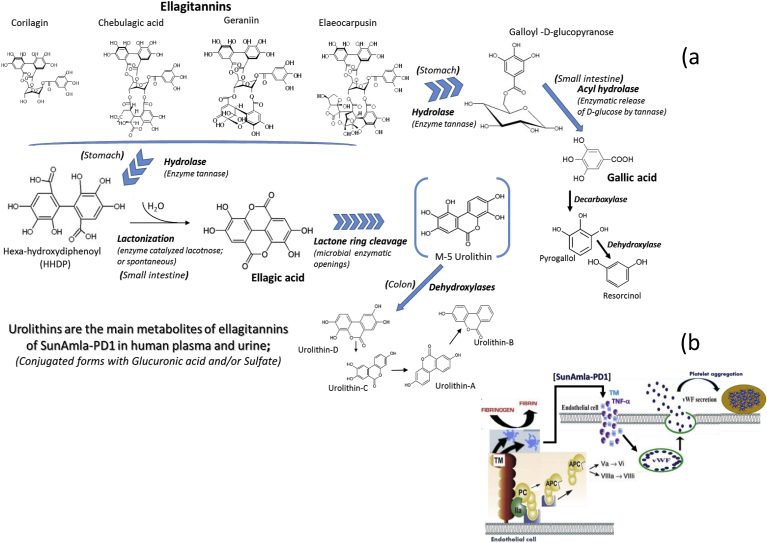
(a) Metabolic pathways of amla: Urolithins are the major tentative metabolites of ellagitannins of amla formulation associated with different health benefits, and (b) Amla inhibits the vWF stimulated thrombin (TM) generation by the reduction in vWF secretion.

Vegetables and fruits form essential elements of a healthy diet; however, ellagitannins and ellagic acid are the polyphenol often underestimated in our diets [20,21]. Interest in ellagitannins is still growing [22]; therefore, it is important to identify safer and more cost-effective strategies for managing common health issues. The present study was designed to evaluate the safety and efficacy of ellagitannins (hydrolyzable to ellagic acid and gallic acid) rich amla (500 mg/day) compared to a placebo in healthy humans. Risk factors such as hypertension, dyslipidemia, and atherosclerosis are emerging as critical components associated with the pathophysiology of accelerated impaired endothelial functions [2,12,13]. The oxidative stress in the vascular endothelium cell is primarily associated with thrombosis, while increased platelet aggregation is yet another significant risk factor for cardiovascular diseases [[23], [24], [25]]. The high blood lipid profiles and platelet aggregation in blood vessels can lead to hypertension. Inhibition of platelet aggregation by oral supplementation of amla is attributable primarily to the abundance of low molecular weight (<1000 Da) hydrolysable ellagitannins (i.e. chebulagic acid, pedunculagin, geraniin, corilagin, elaeocarpusin, etc.), gallic acid, ellagic acid, and their metabolites which are illustrated in Fig. 3a. Hydrolysis of ellagitannins yields galloyl-glucose residues, which are eventually transformed into gallic acid, pyrogallol and resorcinol [26,27], along with HHDP moiety, which undergoes lactonization and spontaneous rearrangement to stable ellagic acid. Due to very poor bioavailability of ellagitannins, they are found relatively low concentrations in the gastrointestinal (GI) tract including feces; therefore the ellagitannins have not been reported in the human systematic circulation system or urine. Urolithins are the primary metabolites of ellagitannins of amla in human plasma and urine mostly present as a conjugated form with glucuronic acid or sulfate. Urolithin-A metabolite was recently reported to possess several biological activities such as anti-inflammatory effects [27]. Thus, ellagitannins of amla are associated with health benefits that are mediated by the bioconversion of ellagitannins by gut microbiota.

Earlier studies demonstrated the beneficial antioxidant activity and cytoprotective effect of amla formulations on dyslipidemia and lipid peroxidation [23,28]. Amla has an anti-thrombotic and anti-oxidative effect. It may also help control vascular injuries in humans due to hyperglycemia that has been positively associated with oxidative stress, which is one of the principal mechanisms involved in the damage of endothelial function. In this study, amla supplementation showed a beneficial effect on endothelial function by modulating thrombin and endothelial vWF, along with a significant improvement in biomarkers of oxidative stress, including TBARS and 8-OHdG. The vWF is a critical adhesive protein that captures platelets from circulation, which usually changes to an elongated form that provides an adhesive surface for platelets via binding to glycoprotein [29]. Oral supplementation of amla inhibits the vWF stimulated thrombin generation by the reduction of vWF secretion in the circulatory system and, thus prevent the vWF mediated adhesion of platelets to fibrin (Fig. 3b), which is a prerequisite for the thrombin to expose anionic phospholipids at the plasma membrane of platelets to facilitate the adhesion. The vWF appears to be an essential factor in the generation of thrombin and subsequent conversion of plasma protein fibrinogen into fibrin [30]. Therefore, amla intake reduces the thrombin formation in vWF deficient platelet-rich plasma (Fig. 3b and c). The oral administration of amla reduced TBARS levels in plasma, suggesting amla consumption could also ameliorate oxidative stress due to aging-related mitochondrion dysfunction. A significant decrease in the cellular oxidative stress biomarker 8-OHdG excretion, an oxidized nucleoside of DNA, represents a decrease in oxidative DNA damage as a result of amla supplementation compared to the placebo. The overall ability to reduce oxidation induced DNA damage and repair of DNA lesions is believed to depend on the time phase relationship. However, an influence of metabolic rate (oxygen consumption) on the 8-OHdG excretion cannot be excluded due to its standard dual function in base excision repair (BER; the cellular mechanism that repairs damaged DNA) as well as in the oxidation of DNA [31]. Further, it is also postulated that metabolites of ellagitannins and polyphenols of amla could be radially adsorbed by cell tissues during regular supplementation, and thus enhancing antioxidant protection against oxidation-induced DNA damage and mutation.

Although there is minimal human evidence on amla in the scientific literature, it appears to be very promising as it could lower blood glucose in both healthy people and diabetics [6]. In animal research, amla appears to be able to reduce triglycerides, improve the cholesterol profile, and benefit cardiovascular health. Most of these actions are attributed to its antioxidant properties, which are partially derived from a reasonably high vitamin C content in addition to the ellagitannins. Amla is beneficial in preventing age-related kidney diseases when administration resulted in significantly decreased levels of lipids [32]. Benefits appear to extend to humans, and the present study demonstrated that four weeks of oral supplementation of standardized amla effectively alters the lipid levels by decreasing LDL cholesterol, total triglycerides, and total cholesterol levels along with a concomitant increase of HDL cholesterol, whereas placebo did not have any considerable effect on any of the study parameters. The results were comparable to studies reported previously [32]. Lipid-lowering properties and endothelial function management activity of amla may be attributed to the polyphenol content and ellagitannins of the formulation. It is also postulated that the ellagitannins present in amla delay the oxidation of vitamin C, while the synergic effect of pectin has also been reported to decrease the cholesterol levels in humans [12] along with the flavonoid components of amla are mainly responsible for a potent hypolipidemic effect. Although several mechanisms [5,33] such as interference with cholesterol absorption, an increase in lecithin-cholesterol acyltransferase activity, and inhibition of HMG-CoA reductase activity are reported in literature for the alteration in the lipid profiles upon supplementation of amla formulations; however conclusive pieces of evidence are not yet clear [8]. Further, the decreasing trend of total cholesterol could also be a determinant of the increased blood fluidity, since erythrocyte membrane structure that usually decreases the membrane fluidity in the superficial layer with any significant alteration of membrane cholesterol and TBARS.

Further, the white blood cell counts, a surrogate marker of inflammation, which usually correlates positively with platelet counts showed no evidence on their possible association with aforementioned risk factors indicate the possibility of a shared mechanism that drives a detrimental effect of amla intake on the improvement in the blood rheology [25]. Therefore, it may be suggested that hematocrit and WBC could be the determinants of the blood fluidity a primary efficacy parameter of this study. Moreover, the albumin is also a vital protein regarding blood fluidity as it could have a negative correlation with blood passage, but the measured values of albumin were at normal levels in the present study indicates the possibility of the relevance of a weak plasma lipid concentration for blood fluidity. Thus, a significant increase in blood fluidity with amla compared to placebo could be attributed to soft and balanced oxygen transport to the cells. Also, vascular age derived from the waveform index can reflect vascular resistance, as the pathogenesis of small size vessels may affect the vascular age. Since, hemorheology is estimated using MC-FAN which is an *in vitro* assay that uses artificial blood vessels (lumen measures <10 mm; assumed to correspond to small-sized capillary vessels), and significant post-trial changes observed between amla and placebo intake suggests the improvement in vascular resistance and reduced impairment of hemorheology with increased blood fluidity possibly due to sympathetic activation on vascular functions. The prothrombin time was significantly lowered upon amla intake compared to placebo without significant alteration in APTT (almost no interfering with coagulation complex assembly on phospholipid surface). Also, levels of both PT and APTT remained within the normal range, suggested that amla on limits the platelet aggregation due to reduced thrombin, and help accelerate the anticoagulant activity, which could result from a moderate increase in blood fluidity. No considerable differences were noticed in any blood test item MCV, MCH, and MCHC between placebo and amla groups as changes were small and within the normal range, suggesting that observed changes were not medically relevant. Also, no significant differences were noted in urinalysis findings between placebo and amla groups. Also, the blood tests related to liver and renal/kidney functions (blood urea nitrogen, AST, ALT, γ-GPT, creatinine, uric acid, phosphate, calcium, sodium, potassium, and chloride) were standard in all subjects. Although a few adverse events were noted during the trial, they were reported during the placebo and were common conditions likely unrelated to study participation. No symptoms considered to be a side effect were observed during the amla treatment period. These results indicate the tolerance and safety of a daily 500 mg dose of amla in healthy humans. The findings above indicate that amla and its components (ellagitannins, polyphenols and their metabolites) could be recognized as a potent antioxidant with the determinant function of vascular homeostasis, regulating several physiologic properties, including vascular permeability as well as antithrombotic properties that could prevent the arterial thrombosis.

Despite the positive findings of this study, there were few limitations. The first is MC-FAN can detect somewhat lower platelet aggregation related hemorheological data due to its *in vitro* characteristics that use artificial blood vessels and absence of an influence of vascular factors such as endothelial cells or smooth muscle, which must be considered when interpreting the results. Another limitation that this is a relatively small study of only fifteen subjects and despite a strong study design, a larger study is necessary to confirm the health benefits of amla.

## 5 Conclusions

In this clinical study, we confirm that the proprietary amla formulation showed a significant improvement in endothelial function as well as a reduction in biomarkers of oxidative stress. Also, the results suggest that amla intake may increase plasma antioxidant potential and decrease oxidative stress, which can help promote oxidative homeostasis. All of these benefits are possible without influencing hepatic or renal function, or diabetic indices in healthy humans. Lastly, the results from this human clinical study conclusively established that amla has an acceptable sensory and safety profile while providing enormous potential for the management of a healthy lifestyle.

## References

[1] Saklayen M.G. (2018). The global epidemic of the metabolic syndrome. Curr. Hypertens. Rep..

[2] Suganya N., Bhakkiyalakshmi E., Sarada D.V.L., Ramkumar K.M. (2016). Reversibility of endothelial dysfunction in diabetes: role of polyphenols. Br. J. Nutr..

[3] Vanhoutte P.M. (2009). Endothelial dysfunction. Circ. J..

[4] Dasaroju S., Gottumukkala K.M. (2014). Current trends in the research of Emblica officinalis (Amla): a pharmacological perspective. Int. J. Pharm. Sci. Rev. Res..

[5] Variya B.C., Bakrania A.K., Patel S.S. (2016). Emblica officinalis (Amla): a review for its phytochemistry, ethnomedicinal uses and medicinal potentials with respect to molecular mechanisms. Pharmacol. Res..

[6] Akhtar M.S., Ramzan A., Ali A., Ahmad M. (2011). Effect of Amla fruit (Emblica officinalis Gaertn.) on blood glucose and lipid profile of normal subjects and type 2 diabetic patients. Int. J. Food Sci. Nutr..

[7] Yokozawa T., Kim H.Y., Kim H.J., Okubo T., Chu D.C., Juneja L.R. (2007). Amla (Emblica officinalis Gaertn.) prevents dyslipidaemia and oxidative stress in the ageing process. Br. J. Nutr..

[8] Antony B., Benny M., Kaimal T.N.B. (2008). A pilot clinical study to evaluate the effect of Emblica officinalis extract (Amlamax™) on markers of systemic inflammation and dyslipidemia. Indian J. Clin. Biochem..

[9] Zhang Y.J., Abe T., Tanaka T., Yang C.R., Kouno I. (2001). Phyllanemblinins A− F, new ellagitannins from Phyllanthus emblica. J. Nat. Prod..

[10] Khanna S., Das A., Spieldenner J., Rink C., Roy S. (2015). Supplementation of a standardized extract from Phyllanthus emblica improves cardiovascular risk factors and platelet aggregation in overweight/class-1 obese adults. J. Med. Food.

[11] Fatima N., Hafizur R.M., Hameed A., Ahmed S., Nisar M., Kabir N. (2017). Ellagic acid in Emblica officinalis exerts anti-diabetic activity through the action on β-cells of pancreas. Eur. J. Nutr..

[12] Usharani P., Fatima N., Muralidhar N. (2013). Effects of Phyllanthus emblica extract on endothelial dysfunction and biomarkers of oxidative stress in patients with type 2 diabetes mellitus: a randomized, double-blind, controlled study. Diabetes, Metab. Syndrome Obes. Targets Ther..

[13] Rao T.P., Okamoto T., Akita N., Hayashi T., Kato-Yasuda N., Suzuki K. (2013). Amla (Emblica officinalis Gaertn.) extract inhibits lipopolysaccharide-induced procoagulant and pro-inflammatory factors in cultured vascular endothelial cells. Br. J. Nutr..

[14] Fatima N., Pingali U., Pilli R. (2014). Evaluation of Phyllanthus emblica extract on cold pressor induced cardiovascular changes in healthy human subjects. Pharmacogn. Res..

[15] Reddy V.D., Padmavathi P., Kavitha G., Gopi S., Varadacharyulu N. (2011). Emblica officinalis ameliorates alcohol-induced brain mitochondrial dysfunction in rats. J. Med. Food.

[16] Tasanarong A., Kongkham S., Itharat A. (2014). Antioxidant effect of Phyllanthus emblica extract prevents contrast-induced acute kidney injury. BMC Complement Altern. Med..

[17] Chen T.S., Liou S.Y., Chang Y.L. (2009). Supplementation of Emblica officinalis (Amla) extract reduces oxidative stress in uremic patients. Am. J. Chin. Med..

[18] Shivananjappa M.M., Joshi M.K. (2012). Influence of Emblica officinalis aqueous extract on growth and antioxidant defense system of human hepatoma cell line (HepG2). Pharm. Biol..

[19] Kamada H., Okamoto T., Hayashi T., Suzuki K. (2010). An in vitro method for screening anti-platelet agents using a microchannel array flow analyzer. Biorheology.

[20] Ito H. (2011). Metabolites of the ellagitannin geraniin and their antioxidant activities. Planta Med..

[21] Tomas-Barberan F.A., García-Villalba R., Gonzalez-Sarrias A., Selma M.V., Espin J.C. (2014). Ellagic acid metabolism by human gut microbiota: consistent observation of three urolithin phenotypes in intervention trials, independent of food source, age, and health status. J. Agric. Food Chem..

[22] Garcia-Muñoz C., Vaillant F. (2014). Metabolic fate of ellagitannins: implications for health, and research perspectives for innovative, functional foods. Crit. Rev. Food Sci. Nutr..

[23] Rao T.P., Htay H.H., Yasuda N.K., Sugino H., Ohkubo T., Okamoto T., Suzuki K. (2014). Antioxidant and anti-thrombotic properties of selected plant extracts of Asia. Austin J. Nutr. Metab..

[24] Kimura T., Inamizu T., Sekikawa K., Kakehashi M., Onari K. (2009). Determinants of the daily rhythm of blood fluidity. J. Circadian Rhythms.

[25] Schmid-Schönbein H., Rieger H., Fischer T. (1980). Blood fluidity as a consequence of red cell fluidity: flow properties of blood and flow behavior of blood in vascular diseases. Angiology.

[26] Mingshu L., Kai Y., Qiang H., Dongying J. (2006). Biodegradation of gallotannins and ellagitannins. J. Basic Microbiol..

[27] Larrosa M., García-Conesa M.T., Espín J.C., Tomás-Barberán F.A. (2010). Ellagitannins, ellagic acid and vascular health. Mol. Asp. Med..

[28] Antony B., Merina B., Sheeba V. (2008). Amlamax™ in the management of dyslipidemia in humans. Indian J. Pharm. Sci..

[29] Mammadova-Bach E., Ollivier V., Loyau S., Schaff M., Dumont B., Favier R., Mangin P.H. (2015). Platelet glycoprotein VI binds to polymerized fibrin and promotes thrombin generation. Blood.

[30] Miszta A., Pelkmans L., Lindhout T., Krishnamoorthy G., de Groot P.G., Hemker C.H., de Laat B. (2014). Thrombin-dependent incorporation of von Willebrand factor into a fibrin network. J. Biol. Chem..

[31] Halliwell B. (1999). Antioxidant defence mechanisms: from the beginning to the end (of the beginning). Free Radic. Res..

[32] Yokozawa T., Kim H.Y., Kim H.J., Tanaka T., Sugino H., Okubo T., Juneja L.R. (2007). Amla (Emblica officinalis Gaertn.) attenuates age-related renal dysfunction by oxidative stress. J. Agric. Food Chem..

[33] Yadav V., Duvey B., Sharma S., Devi B. (2014). Amla (emblica officinalis)–medicinal food and pharmacological activity. Int. J. Pharm. Chem. Sci..

