# Supplementary material for: Clinical evaluation of Emblica Officinalis Gatertn (Amla) in healthy human subjects: Health benefits and safety results from a randomized, double-blind, crossover placebo-controlled study
Source: Contemp Clin Trials Commun. 2019 Nov 27;17:100499. doi: 10.1016/j.conctc.2019.100499 (PMC6926135; doi:10.1016/j.conctc.2019.100499)
Supplement: Multimedia component 1 [file mmc1.pdf]

Kapoor et al. (Supplementary Information; Contemporary Clinical Trials Communications; j.conctc.2019.100499)

Table ST1. Efficacy of amla on blood biochemical assessment and lipid profiles compared to placebo among the healthy human subjects.

Parameters	Placebo intake (mean± sem)				Amla intake (mean± sem)				ANCOVA (Among Groups)
	Before (W1, W2) or (W10, W11)	2 Weeks (W3, W4) or (W12, W13)	4 Weeks (W5, W6) or (W14, W15)	Post (3 Weeks) (W7,W9,W9) or (W16,W17,W18)	Before (W1, W2) or (W10, W11)	2 Weeks (W3, W4) or (W12, W13)	4 Weeks (W5, W6) or (W14, W15)	Post (3 Weeks) (W7,W9,W9) or (W16,W17,W18)	
Glucose Fasting (mg/dL)	93.6± 3.06	92.2± 2.20	95.2 ± 2.45	94.7 ± 3.67	92.5 ±2.75	93.8 ± 2.76	95.7 ± 2.63	95.3 ± 2.44	2W: F=1.61; P= 0.21<br>4W: F=0.41; P= 0.53<br>Post:: F=0.19; P= 0.67
		F=0.51; P= 0.49	F=0.37; P= 0.55	F=0.13; P= 0.72		F=0.73; P= 0.41	F=6.21; P= 0.03*	F=4.30; P= 0.06*	
TG (mg /dL)	183.0±17.8	177.2±12.9	175.8 ±19.0	165.8±15.8	170.5± 12.3	182.4± 10.7	179.5 ± 19.6	177.8 ± 19.4	2W: F=2.05; P= 0.17<br>4W: F=1.58; P= 0.22<br>Post:: F=2.06; P= 0.17
		F=0.10; P= 0.76	F=0.08; P= 0.78	F=4.26; P= 0.06		F=4.23; P= 0.06	F=2.63; P= 0.13	F=0.80; P= 0.39	
TC (mg /dL)	240.0 ±7.65	233.9± 10.33	238.7 ± 8.51	238.0 ± 10.6	237.2±9.06	227.3±8.42	235.8 ± 9.08	232.8 ± 7.2	2W: F=0.47; P= 0.50<br>4W: F=0.0; P= 1.0<br>Post:: F=0.13; P= 0.72
		F=2.05; P= 0.18	F=0.12; P= 0.74	F=0.06; P= 0.81		F=2.66; P= 0.13	F=0.07; P= 0.80	F=0.44; P= 0.52	
HDL-Cho (mg /dL)	48.5 ±1.81	49.3±2.47	49.5±2.00	49.5±1.69	47.5±1.83	49.8±1.78	48.2±1.82	49.2± 1.79	2W: F=5.22; P= 0.03*<br>4W: F=1.99; P= 0.17<br>Post:: F=0.73; P= 0.40
		F=0.47; P= 0.51	F=0.56; P= 0.47	F=0.70; P= 0.42		F=8.06; P= 0.02*	F=2.25; P= 0.16	F=3.26; P= 0.09	
LDL-Cho (mg /dL)	156.4±6.55	157.5±6.99	155.6±6.56	155.6±8.37	157.2±7.44	145.3±5.83	146.2±6.55	150.2±6.87	2W: F=2.95; P= 0.10<br>4W: F=1.45; P= 0.24<br>Post:: F=0.54; P= 0.47
		F=0.05; P= 0.83	F=0.01; P= 0.92	F=0.01; P= 0.92		F=3.35; P= 0.09	F=1.58; P= 0.23	F=2.57; P= 0.13	
HbA1c (%)	5.45±0.0.08	5.50 ±0.09	5.52±0.10	5.40 ±0.09	5.41 ±0.09	5.42 ±0.07	5.45 ±0.07	5.42 ±0.07	2W: F=0.18; P= 0.68<br>4W: F=0.0; P= 1.0<br>Post:: F=0.49; P= 0.49
		F=1.32; P= 0.27	F=1.78; P= 0.21	F=1.05; P= 0.33		F=0.03; P= 0.87	F=0.49; P= 0.50	F=0.02; P= 0.89	
Na (mEq /L)	141.6 ±0.40	142.2±0.30	141.4 ±0.37	141.5±0.45	142.3 ±0.41	142.1 ±0.21	141.5 ±0.24	141.8 ± 0.42	2W: F=0.65; P= 0.43<br>4W: F=0.33; P= 0.57<br>Post:: F=0.34 ; P= 0.57
		F=2.56; P= 0.14	F=0.32; P= 0.58	F=0.04; P=0.84		F=5.66; P= 0.04*	F=0.45; P= 0.52	F=1.06; P= 0.32	
K *(mEq /L)*	4.43±0.08	4.50 ±0.09	4.37 ±0.08	4.52±0.11	4.27 ±0.09	4.56 ±0.08	4.64 ±0.12	4.44 ±0.13	2W F=7.65; P= 0.011*:<br>4W: F= 8.54; P= 0.008*<br>Post:: F=0.9; P= 0.35
		F=0.86; P= 0.37	F=0.79; P= 0.39	F=1.10; P= 0.31		F=20.5; P= 0.001*	F=11.2; P= 0.006*	F=3.31; P= 0.09	
Cl (mEq /L)	101.5±0.43	102.2 ±0.41	102.7±0.54	103.4 ±0.51	101.9 ±0.49	103.0 ±0.48*	103.7 ±0.52	103.4 ±0.63	2W: F=1.58; P= 0.22<br>4W: F=3.95; P= 0.05*<br>Post:: F= 0.61; P= 0.44
		F=8.53; P= 0.01*	F=15.2; P= 0.002*	F=54.9; P= <0.001*		F=27.0; P= <0.001*	F=22.4; P= <0.001*	F=19.2; P= <0.001*	
Ca (mg /dL)	9.38±0.09	9.39 ±0.10	9.40±0.10	9.26±0.09	9.30 ±0.10	9.38 ±0.08	9.34 ±0.09	9.26 ±0.09	2W: F=0.75; P= 0.40<br>4W: F=0.08; P= 0.78<br>Post:: F= 0.05; P= 0.83
		F=0.01; P= 0.92	F=0.03; P= 0.87	F=2.74; P= 0.12		F=2.01; P= 0.18	F=0.25; P= 0.63	F=0.14; P= 0.71	
P (mg /dL)	3.21 ±0.14	3.07 ±0.16	3.02 ±0.14	3.25 ±0.15	3.11±0.16	3.15 ±0.13	3.32±0.18	3.30 ±0.14	2W: F=1.43; P= 0.24<br>4W: F=6.02; P= 0.022*<br>Post: F= 0.95; P= 0.34:
		F=2.15; P= 0.17	F=6.21; P= 0.03*	F=0.2; P= 0.66		F=0.16; P= 0.70	F=9.31; P= 0.01*	F=6.79; P= 0.02*	

Table ST2. Evaluation of activities of hepatic risk parameters to assess the possible changes in liver function and related safety aspects during amla supplementation among the healthy human subjects.

**Parameters**	**Placebo intake (mean± sem)**				**Amla intake (mean± sem)**				**ANCOVA (Among Groups)**
	Before (W1, W2) or (W10, W11)	2 Weeks (W3, W4) or (W12, W13)	4 Weeks (W5, W6) or (W14, W15)	Post (3 Weeks) (W7,W9,W9) or (W16,W17,W18)	Before (W1, W2) or (W10, W11)	2 Weeks (W3, W4) or (W12, W13)	4 Weeks (W5, W6) or (W14, W15)	Post (3 Weeks) (W7,W9,W9) or (W16,W17,W18)	
Total Protein (g/dL)	7.62 ±0.11	7.55 ± 0.10	7.50 ± 0.11	7.43 ± 0.15	7.52± 0.09	7.50 ± 0.08	7.40 ± 0.05	7.49 ± 0.11	2W: F=0.05; P= 0.83<br>4W: F=0.2; P= 0.66<br>Post: F=1.26; P= 0.27
		F=1.75; P= 0.21	F=3.10; P= 0.10	F=15.6; P= 0.002**		F=0.03; P= 0.87	F=3.61; P= 0.08	F=0.08; P= 0.78	
Albumin (g /dL)	4.53±0.09	4.51±0.08	4.51 ±0.07	4.45±0.07	4.49± 0.07	4.45± 0.06	4.45 ± 0.07	4.50 ± 0.09	2W: F=0.07; P= 0.79<br>4W: F=0.05; P= 0.83<br>Post: F=1.69; P= 0.21:
		F=0.68; P= 0.43	F=0.46; P= 0.51	F=6.29; P= 0.03*		F=0.51; P= 0.49	F=0.38; P= 0.55	F=0.08; P= 0.78	
Albumin/Globulin (A/G Ratio)	1.48 ±0.05	1.49± 0.05	1.52 ± 0.05	1.51 ±0.04	1.49±0.05	1.48±0.04	1.52 ± 0.05	1.52 ± 0.06	2W: F=0.34; P= 0.57<br>4W: F=0.03; P= 0.86<br>Post: F=0.01; P= 0.92
		F=0.08; P= 0.78	F=3.21; P= 0.10	F=0.88; P= 0.37		F=0.31; P= 0.59	F=0.99; P= 0.34	F=0.51; P= 0.49	
AST (GOT) (IU /L /37° C)	21.5 ±1.04	21.2±1.35	22.5±1.42	22.8±2.40	21.1±1.31	21.3±1.31	21.7±1.29	21.7± 1.02	2W: F=0.14; P= 0.71<br>4W: F=0.0; P= 1.0<br>Post: F=0.0; P= 1.0
		F=0.2; P= 0.66	F=1.18; P= 0.30	F=0.58; P= 0.46		F=0.03; P= 0.87	F=0.35; P= 0.57	F=0.81; P= 0.39	
ALT(GPT)m (IU /L /37° C)	26.9±3.33	26.5±4.27	27.5±4.12	27.7±3.64	29.5±3.76	25.9±3.91	28.0±3.10	28.4±4.30	2W F=1.64; P= 0.21:<br>4W: F=0.38; P= 0.54<br>Post: F=0.53; P= 0.47
		F=0.02; P= 0.89	F=0.07; P= 0.80	F=0.37; P= 0.55		F=8.06; P= 0.02*	F=0.47; P= 0.51	F=0.22; P= 0.65	
ALP (IU /L /37° C)	257.2±17.3	251.7 ± 16.8	249.5 ± 16.7	256.1 ± 17.9	245.2±15.3	244.5±19.0	242.1±17.6	259.0±0.17.8	2W: F= 0.18; P= 0.68<br>4W: F=0.15 ; P= 0.70<br>Post: F=0.90; P= 0.35
		F=0.73; P= 0.41	F=1.33; P= 0.27	F=0.01; P=0.92		F=0.01; P= 0.92	F=0.46; P= 0.51	F=2.5; P= 0.14	
γ-GPT (IU /L /37° C)	45.3 ±5.61	43.2 ±5.50	43.3 ±5.63	46.5 ± 8.58	42.0 ±5.45	39.9±5.21	40.4 ±5.18	41.2±4.58	2W: F=0.01; P= 0.92<br>4W: F=0.0; P= 1.0<br>Post: F=0.12 ; P= 0.73
		F=0.84; P= 0.38	F=0.91; P= 0.36	F=0.04; P= 0.84		F=0.48; P= 0.50	F=0.56; P= 0.47	F=0.11; P=0.75	
Creatinine (mg /dL)	0.70±0.04	0.69 ±0.04	0.70±0.04	0.70 ±0.04	0.70 ±0.04	0.71 ±0.04	0.72 ±0.04	0.71 ±0.04	2W: F=0.18; P= 0.66<br>4W: F=0.09; P= 0.77<br>Post: F= 0.32; P= 0.58
		F=0.15; P= 0.71	F=0.0; P= 1.0	F=0.25; P= 0.63		F=4.65; P= 0.05*	F=7.88; P= 0.02*	F=4.65; P= 0.05*	
Uric Acid (mg /dL)	6.26±0.39	6.41 ±0.47	6.50±0.44	6.10±0.33	6.14 ±0.36	6.32 ±0.46	6.25 ±0.39	6.30 ±0.37	2W: F=0.04; P= 0.84<br>4W: F=0.16; P= 0.69<br>Post: F= 1.44; P= 0.24
		F=0.54; P= 0.47	F=1.0; P= 0.34	F=0.56; P= 0.47		F=0.86; P= 0.37	F=0.59; P= 0.46	F=1.42; P= 0.26	
Urea-Nitrogen (mg /dL)	13.6±0.74	13.3 ±0.65	14.2±0.76	13.2 ±0.50	12.7 ±0.51	13.4 ±0.1.0	13.5 ±0.56	14.3 ±0.87	2W: F=0.49; P= 0.49<br>4W: F=0.56; P= 0.46<br>Post: F= 3.12; P= 0.09
		F=0.18; P= 0.68	F=1.03; P= 0.33	F=0.25; P= 0.63		F=0.67; P= 0.42	F=2.04; P= 0.18	F=3.77; P= 0.08	
Total Bilirubin (mg /dL)	0.67±0.0621	0.63 ±0.06	0.64±0.07	0.63 ±0.07	0.71 ±0.0.09	0.55 ±0.07	0.57 ±0.05	0.62 ±0.06	2W: F=1.91; P= 0.18<br>4W: F=1.81; P= 0.19<br>Post: F= 0.51; P= 0.48
		F=0.37; P= 0.55	F=0.21; P= 0.65	F=0.19; P= 0.67		F=5.58; P= 0.04*	F=2.85; P= 0.12	F=2.83; P= 0.12	

Table ST3. Subjective qualitative urinalysis parameters assessment during amla supplementation among the healthy human subjects.

**Parameters**	**Placebo intake (mean± sem)**				**Amla intake (mean± sem)**				**ANCOVA (Among Groups)**
	Before (W1, W2) or (W10, W11)	2 Weeks (W3, W4) or (W12, W13)	4 Weeks (W5, W6) or (W14, W15)	Post (3 Weeks) (W7,W9,W9) or (W16,W17,W18)	Before (W1, W2) or (W10, W11)	2 Weeks (W3, W4) or (W12, W13)	4 Weeks (W5, W6) or (W14, W15)	Post (3 Weeks) (W7,W9,W9) or (W16,W17,W18)	
Specific Gravity (Value)	1.02 ±0.002	1.02 ±0.002	1.02 ±0.002	1.02 ± 0.002	1.02 ±0.002	1.02 ±0.002	1.02 ±0.002	1.02 ± 0.001	2W: F=0.99; P= 0.33<br>4W: F=0.49; P= 0.49<br>Post: F=0.19; P= 0.67
		F=0.0; P= 1.0	F=0.0; P= 1.0	F=0.0; P= 1.0		F=0.0; P= 1.0	F=0.0; P= 1.0	F=0.0; P= 1.0	
pH (Value)	6.0±0.19	6.12±0.30	5.62 ±0.18	6.0±0.27	5.92± 0.28	6.0± 0.29	5.54 ± 0.20	5.69 ± 0.17	2W: F=0.0; P= 1.0<br>4W: F=1.08; P= 0.31<br>Post: F=0.51; P= 0.48
		F=0.11; P= 0.75	F=2.08; P= 0.17	F=0.0; P= 1.0		F=0.08; P= 0.78	F=1.99; P= 0.18	F=0.65; P= 0.44	
Urobilinogen	（±）; N=13	（±）; N=13	（±）; N=13	（±）; N=13	（±）; N=13	（±）; N=13	（±）; N=13	（±）; N=13	
Protein	（－）; N=13	（－）; N=13	（－）; N=13	（－）; N=12,<br>(±); N=1	（－）;N=12,<br>（±）; N=1	（－）; N=13	（－）; N=13	（－）; N=13	
Sugar	（－）; N=13	（－）; N=13	（－）; N=13	（－）; N=13	（－）;N=13	（－）; N=13	（－）;N=13	（－）;N=12,<br>（1＋）; N=1	
Urinary Ketone Body	（－）; N=13	（－）; N=13	（－）; N=13	（－）; N=13	（－）;N=13	（－）; N=13	（－）; N=12,<br>（2＋); N=1	（－）;N=13	
Urinary Occult Blood reaction	（－）; N=9,<br>（±）; N=4	（－）; N=10,<br>（±）; N=3	（－）; N=10,<br>(±); N=3	（－）; N=9,<br>(±); N=4	（－）; N=12,<br>（±）; N=1	（－）; N=11、<br>（±）; N=1、<br>（1＋）; N=1	（－）; N=7,<br>(±); N=6	（－); N=8,<br>(±); N=5	

Table ST4. The mapping of adverse events reported and confirmed acceptability of amla supplementation among the healthy human subjects.

Subject numbers	Placebo intake				Amla intake			
	Before (W1, W2) or (W10, W11)	2 Weeks (W3, W4) or (W12, W13)	4 Weeks (W5, W6) or (W14, W15)	Post (3 Weeks) (W7,W9,W9) or (W16,W17,W18)	Before (W1, W2) or (W10, W11)	2 Weeks (W3, W4) or (W12, W13)	4 Weeks (W5, W6) or (W14, W15)	Post (3 Weeks) (W7,W9,W9) or (W16,W17,W18)
1	**Drop Out** *(after screening)*							
2	None	**Diarrhea Feelings** (W3, W4)	**Diarrhea Feelings** (W5, W6)	**Diarrhea Feelings** (W7,W9,W9)	None	None	None	None
3	None	None	None	None	None	None	None	None
4	None	None	None	None	None	None	None	None
5	None	None	None	None	None	None	None	None
6	None	None	None	None	None	None	None	None
7	None	**Cold** (W3, W4)	**Slightly Cold** (W5,W6)	None	None	None	None	None
8	None	None	None	None	None	None	None	None
9	None	None	None	None	**Edema** (W1,W2)	None	None	None
10	None	None	None	**Drop Out** *(after W6)*				
11	None	None	None	None	None	None	None	None
12	None	**Loose stool** (W3, W4)	None	None	None	None	None	None
13	None	None	None	None	None	None	None	None
14	None	None	None	None	None	None	None	None
15	None	None	**Constipation** (W5, W6)	None	None	None	None	None

None= *No adverse event reported*
